# Tattoo-Associated Sarcoid-like Uveitis: A Multicenter Registry Study

**DOI:** 10.3390/biomedicines14030702

**Published:** 2026-03-18

**Authors:** Ryoji Yanai, Yuko Misaki, Mariko Egawa, Shido Nagaki, Kumi Shirai, Toshikatsu Kaburaki, Suguru Nakagawa, Yukako Hiramatsu, Kinya Tsubota, Yoshihiko Usui, Sho-Hei Uchi, Takanori Aoki, Kenji Nagata, Chie Sotozono, Shiori Kuramoto, Nobuyo Yawata, Koh-Hei Sonoda, on behalf of Multicenter Tattoo-Associated Sarcoid Uveitis Registry institutions in the Japanese Ocular Inflammatory Association

**Affiliations:** 1 Department of Ophthalmology, Tokushima University Hospital, Tokushima 770-8503, Japan; yuko.tabaru@tokushima-u.ac.jp (Y.M.); egawa.m@tokushima-u.ac.jp (M.E.);; 2 Department of Ophthalmology, Kainan Iryou Center, Wakayama 642-0002, Japan; 3 Department of Ophthalmology, Jichi Medical University, Shimotsuke 329-0498, Japan; 4 Department of Ophthalmology, Saitama Medical Center, Jichi Medical University, Saitama 330-8503, Japan; 5 Department of Ophthalmology, Ehime University Graduate School of Medicine, Toon, Ehime 791-0295, Japan; hiramatsu.yukako.bg@ehime-u.ac.jp; 6 Department of Ophthalmology, Tokyo Medical University Hospital, Tokyo 160-8402, Japan; 7 Department of Ophthalmology, Yamaguchi University Hospital, Yamaguchi 755-8505, Japan; 8 Department of Ophthalmology, Kyoto Prefectural University of Medicine, Kyoto 602-8566, Japan; 9 Department of Ophthalmology, Graduate School of Medical Sciences, Kyushu University, Fukuoka 812-8582, Japan

**Keywords:** tattoo, sarcoidosis, uveitis, registry, glaucoma

## Abstract

**Background**: This study aims to characterize the clinical features and outcomes of tattoo-associated sarcoid-like uveitis using a multicenter uveitis registry given the limited existing data. **Design**: This is a retrospective study. **Participants**: Ten patients (20 eyes) diagnosed with tattoo-associated sarcoid-like uveitis took part in the study. **Methods**: The data of patients newly evaluated at participating registry centers from January 2000 to June 2025 were reviewed. Demographic data, treatments, visual acuity, recurrence, glaucoma/intraocular pressure (IOP)-lowering therapy, extraocular involvement, and histologic confirmation were extracted when available. **Main Outcome Measures**: Recurrence, glaucoma/IOP-lowering therapy, extraocular involvement, and change in logarithm of the minimum angle of resolution (logMAR) from presentation to final follow-up were measured. **Results**: Seven (70%) patients were male, and the patients’ mean age was 35.1 ± 7.8 years. All patients exhibited bilateral ocular involvement. Histologic confirmation at the tattoo site was documented in five (50%) patients. The mean logMAR visual acuity was 0.12 ± 0.31 at presentation and 0.16 ± 0.42 at the final follow-up (median follow-up: 20 months). All patients received topical corticosteroids; periocular steroids were administered in seven cases (70%), oral systemic steroids in four (40%), adalimumab in two (20%), and cyclosporin in one (10%). Seven cases (70%) developed uveitis recurrence, and eight received glaucoma/IOP-lowering therapy (80%). Extraocular inflammation affected the skin/tattoo in seven patients (70%) and the axillary lymph nodes in one (10%). This finding is definitive; however, this is also true even when the organ/body part is plural (e.g., lungs). **Conclusions**: Tattoo-associated sarcoid-like uveitis often follows a chronic course with frequent recurrence and uveitic glaucoma. Thus, close ophthalmic monitoring and coordinated systemic evaluation may be warranted.

## 1. Introduction

Tattooing has become increasingly prevalent worldwide, particularly among younger individuals [1,2,3,4], and cutaneous reactions to tattoo pigments range from acute inflammation to chronic granulomatous disease [1]. During tattooing, ink is injected into multiple skin layers, where it becomes fixed within the dermis. The process is intended to temporarily suppress local immune responses, but trace ink particles are transported systemically by circulating white blood cells. A proportion of pigment remains at the injection site through uptake by tissue-resident macrophages and persists via a “capture–release–recapture” mechanism, ensuring long-term visibility [5,6].

Tattoo granuloma with uveitis is recognized as a distinct clinical entity, often overlapping clinically and histopathologically with sarcoidosis [7,8,9,10]. According to prior reports and reviews, tattoo-associated uveitis is commonly bilateral and recurrent, and may be accompanied by systemic sarcoidosis or sarcoid-like inflammation [7,8,9,10,11,12,13,14]. However, the existing evidence is limited to single-center series and case reports, and comprehensive data on the treatment patterns and outcomes remain scarce [8,10]. To address this gap, the present study aimed to explore the clinical spectrum, treatments, recurrence, and glaucoma-related outcomes of tattoo-associated sarcoid-like uveitis in Japan using a national coverage-multicenter uveitis registry.

## 2. Materials and Methods

Study design and setting: We conducted a retrospective analysis of the data obtained from patients enrolled in a national coverage-multicenter uveitis registry. Patients newly presenting to the participating registry centers from January 2000 to June 2025 were included.

Definition: In the present study, tattoo-associated sarcoid-like uveitis was defined as follows: (1) uveitis with granulomatous features clinically consistent with ocular sarcoidosis; (2) a temporal association with tattoo inflammation or pigment-related cutaneous findings; and (3) physician attribution of uveitis to tattoo-associated sarcoid-like disease.

Data collection: Demographic data (age and sex), laterality, laboratory results, extraocular involvement, treatments (topical corticosteroids, periocular corticosteroid injections, systemic corticosteroids, and immunomodulatory therapy), and clinical outcomes were extracted from the patients’ medical records. Missing data were assessed for each variable. The analyses were primarily conducted using available-case (pairwise) data; the denominator, therefore, varies across analyses depending on data availability. No imputation was performed because missingness reflected incomplete documentation in routine clinical care.

Outcome measures: The primary outcomes included uveitis recurrence, glaucoma/intraocular pressure (IOP)-lowering therapy, extraocular involvement, and change in logarithm of the minimum angle of resolution (logMAR) visual acuity from presentation to final follow-up, with visual acuity analyzed at the eye level (20 eyes). Uveitis recurrence was defined as disease activity requiring the initiation or escalation of anti-inflammatory treatment, and glaucoma/IOP-lowering therapy was defined as the need for the initiation or escalation of IOP-lowering treatment.

Statistical analysis: Continuous variables are presented as means ± standard deviations or medians (interquartile ranges), whereas categorical variables are provided as counts (percentages).

## 3. Results

**Figure 1:**
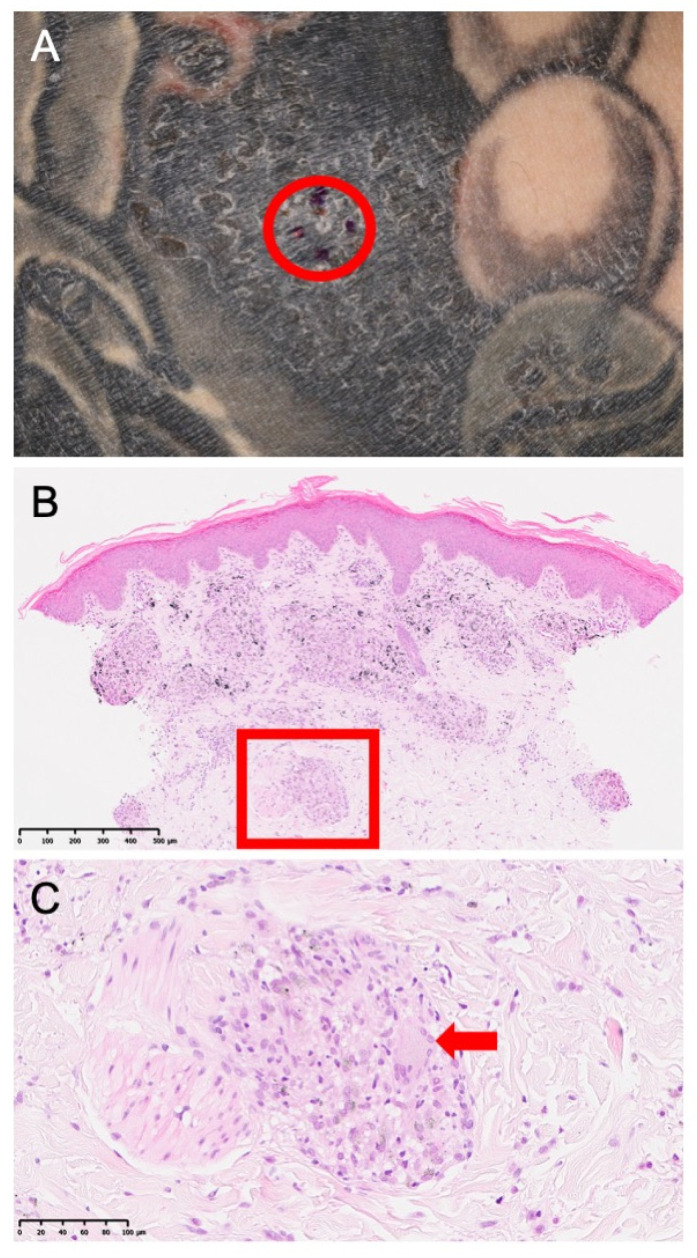
A patient’s tattooed skin, punch biopsy, and histopathology. (**A**) Right upper arm showing infiltration and scaling limited to the intensely pigmented black area. A punch biopsy sample was obtained from the marked site (red circle). (**B**) Hematoxylin-and-eosin-stained section of the biopsy (original magnification: ×50) showing multiple foci in the upper to mid dermis. The region outlined by the red box is shown in panel (**C**). (**C**) A higher power view (original magnification: ×200) showing black pigment deposits surrounded by epithelioid cell granulomas with minimal lymphocytic infiltration. Langhans-type giant cells with peripheral, horseshoe-shaped nuclei are visible (red arrow).

Ten patients (20 eyes) met the inclusion criteria, representing 100% of the patients enrolled in the registry (Table 1). Among these, seven (70%) were male, and the mean age was 35.1 ± 7.8 (27–44) years. The median follow-up duration was 19.1 (interquartile range: 8.3–26.0) months. Histologic confirmation at the tattoo site was obtained in four patients (40%, Figure 1). Bilateral hilar lymphadenopathy was not observed in any case (0/10). Elevated angiotensin-converting enzyme and elevated soluble IL-2 receptor levels were documented in 2/10 and 4/10 cases, respectively (Table 2). T-spot testing was negative in patients in whom it was performed, with several cases not assessed (Table 2). Data on latency from tattooing to uveitis onset, which ranged from 1 to 29 years, were available in four cases (Table 2).

**Figure 2:**
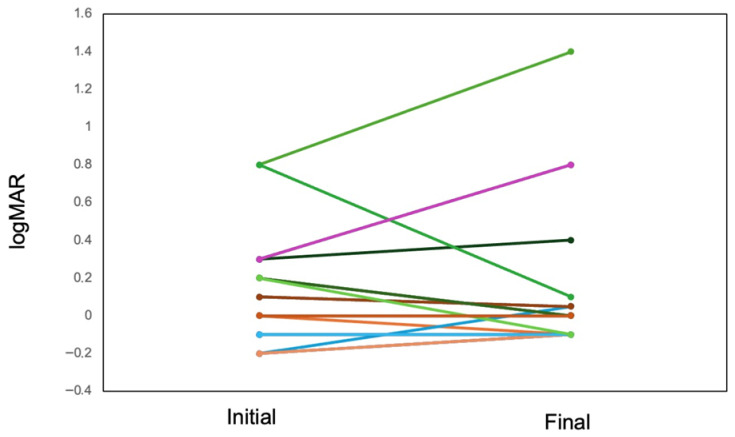
Change in visual acuity at the eye level from the initial visit to the final visit.

**Figure 3:**
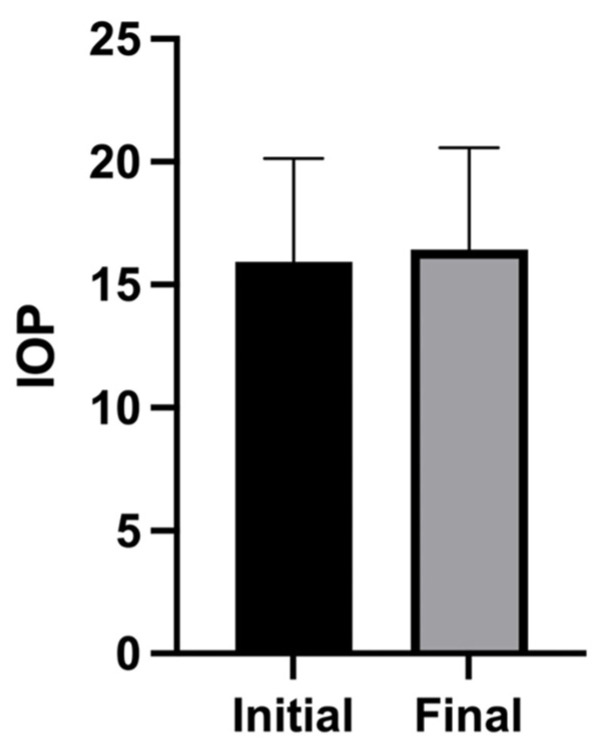
Change in intraocular pressure from the initial visit to the final visit.

At the eye level, the mean logMAR visual acuity across 20 eyes was 0.12 at presentation and 0.15 at the final follow-up (Figure 2). Consistent with this, the patient-level mean logMAR values showed a limited overall change between baseline and final follow-up across individual cases (Table 2). The IOP changed from 14.5 ± 4.1 mmHg (mean ± standard deviation) at the initial visit to 17.6 ± 4.0 mmHg at the final visit (Figure 3). Macular edema was frequently observed, occurring bilaterally in seven patients and unilaterally (left eye) in one patient, affecting a total of 15 out of 20 eyes (75%) (Table 2). Anterior segment inflammation (anterior chamber inflammatory cells) was frequently observed. Vitreous involvement—manifesting as clumped and/or diffuse vitreous opacities—was also common. Posterior segment vasculitic features, including retinal periphlebitis, perivascular nodules, and candle-wax-like retinochoroidal exudates, were variably present. Granulomatous lesions involving the optic disc and/or choroid were noted in a subset of eyes. Structural complications, including tent-shaped/trapezoidal peripheral anterior synechiae and photocoagulation-like retinochoroidal atrophic lesions, were also observed in some cases (Table 3). All cases in the present study had bilateral glaucoma associated with uveitis. Etiologically, 70% of the patients were classified as having glaucoma related to sarcoidosis/uveitis, 50% as having steroid-induced glaucoma, and 40% as having mixed (overlapping) mechanisms. Recurrence occurred in seven cases (70%), and glaucoma/IOP-lowering therapy was required in eight cases (80%) (Table 4). Extraocular inflammation affected the skin/tattoo in seven patients (70%) and the axillary lymph nodes in one (10%) patient.

All patients received topical corticosteroids. Periocular corticosteroid injections were administered in seven patients (70%), oral systemic corticosteroids in four (40%), cyclosporin in one (10%), and adalimumab in two (20%) (Table 4 and Table 5).

## 4. Discussion

A key strength of the present multicenter registry study is its presentation of a comprehensive case series, which included substantially more patients in the analysis compared with prior reports. Tattoo-associated sarcoid-like uveitis was consistently bilateral and frequently recurrent, with over half of the patients requiring IOP-lowering therapy. These findings align with previous case series and comparative reviews, which describe a chronic course and an overlap with systemic sarcoidosis or sarcoid-like granulomatous inflammation [8,9,10].

The high rates of recurrence and glaucoma/IOP-lowering therapy provision suggest that ongoing inflammation can cause secondary structural complications. The proposed mechanisms include persistent antigenic stimulation by tattoo pigments and delayed hypersensitivity or foreign-body granulomatous reactions, potentially extending from the skin to the ocular tissues [10,15]. Elevated IOP was attributed to chronic inflammation in three eyes, steroid-induced glaucoma in one eye, and both mechanisms in four eyes. These findings also indicate that persistent ocular inflammation is a key feature of tattoo-associated sarcoid-like disease and highlight the need for careful differentiation between inflammation-driven and steroid-induced IOP elevations in clinical management.

Notably, not all tattoos pose the same health risks. In traditional Japanese hand-poked tattooing (tebori), famously depicted in Jun-Ichirō Tanizaki’s debut novella Shisei (“The Tattooer”), carbon-based sumi ink, which is regarded as less allergenic owing to carbon’s relative biocompatibility, is often used. Contrarily, modern machine tattoos may incorporate chemically diverse pigments, including azo dyes, quinacridones, phthalocyanines for vivid blue and green hues, and inorganic pigments (e.g., iron oxides and titanium dioxides). These mixtures have been implicated in hypersensitivity reactions and may pose infectious and systemic safety risks [16,17,18,19].

Beyond pigment composition, experimental and ex vivo human studies have indicated that tattoo pigments and trace-element impurities are not confined to the dermis. Using synchrotron-based analyses, colored pigments and associated metals were detected in the regional lymph nodes of tattooed donors, providing evidence of translocation via the lymphatic pathways. Although the clinical significance of such deposition remains unclear, pigment accumulation in the lymph nodes could promote chronic immune activation, complicate lymphadenopathy interpretation, and contribute to sarcoid-like granulomatous responses in predisposed individuals [20].

Most patients were treated with topical and periocular corticosteroids, whereas systemic corticosteroids and biologic therapies were used selectively. Given the potential for systemic involvement, including lymphadenopathy and pulmonary disease, as reported previously [3,10,12], coordinated evaluation with dermatology and internal medicine may be warranted when tattoo-associated inflammation occurs alongside uveitis [21].

The present study has several limitations, including the small sample size, variable data completeness across centers, and lack of standardized, detailed phenotyping, such as uniform anterior chamber cell grading or imaging biomarkers. However, its multicenter registry design provides pragmatic real-world data on the treatment patterns and clinically meaningful outcomes of this rare condition. From a clinical perspective, our findings underscore the importance of considering the presence of tattoo-associated sarcoid-like disease in patients with recurrent uveitis, particularly when ocular flares coincide with inflammation or swelling of the tattooed skin and support the need for coordinated ophthalmologic and systemic evaluations to guide the diagnosis and management of this condition. Another limitation is that the patients’ visual acuity was analyzed at the eye level, whereas the other outcomes were evaluated at the patient level. Although clinically appropriate for bilateral disease, this difference in analytic units may introduce bias in interpretation.

## Figures and Tables

**Table 1:** Baseline characteristics and systemic findings.

Characteristic	Value
No. of patients	10
No. of eyes	20
Age, years	35.1 ± 7.8
Male sex, *n* (%)	7 (70%)
Follow-up, months	19.1 (8.3–26.0)
Bilateral involvement, *n* (%)	10 (100%)
Histological confirmation (including tattoo biopsy), *n* (%)	4 (40%)
Prior systemic sarcoidosis, *n* (%)	3 (30%)
Initial visual acuity, logMAR (eyes)	0.12
Final visual acuity, logMAR (eyes)	0.15

Data are means ± standard deviations, medians (interquartile ranges), or counts (%). Visual acuity data were collected at the eye level. Abbreviation: logMAR, logarithm of the minimum angle of resolution.

**Table 2:** Patient-level demographics and visual acuity.

Case No.	Age	Sex	Latency from Tattooing to Uveitis Onset (Year)	Histology Confirmed	Extraocular Involvement	BHL	ACE	sIL-2R	T-Spot	Initial Mean logMAR	Final Mean logMAR	Macular Edema (Eyes)
1	40	Male	Unknown	Yes	Skin	No	Yes	Yes	No	0.2	0.43	Both
2	34	Male	Unknown	Yes	Skin	No	No	No	No	−0.05	−0.1	
3	49	Female	29	No	None	No	No	No	No	0.2	0.0	Both
4	31	Male	1	No	Skin	No	No	No	No	−0.2	−0.02	Both
5	44	Female	Unknown	No	None	nm	nm	No	No	−0.2	−0.1	
6	28	Male	Unknown	No	Axillary LN	No	No	Yes	No	0.8	0.75	Left
7	27	Male	7	Yes	Skin	No	No	No	No	−0.1	−0.1	Both
8	40	Male	Unknown	Yes	Skin	No	Yes	Yes	No	0.2	0.43	Both
9	26	Male	Unknown	No	Skin	No	nm	Yes	No	0.25	0.15	Both
10	32	Female	10	No	Skin	No	No	No	nm	0.7	0.2	Both

Abbreviations: ACE: angiotensin-converting enzyme, BHL: bilateral hilar lymphadenopathy, LN: lymph node, logMAR: logarithm of the minimum angle of resolution, nm: not measured, sIL-2R: soluble interleukin-2 receptor.

**Table 3:** Clinical findings in an eye diagnosed with tattoo-associated sarcoid-like uveitis.

Case	Mutton-Fat Keratic Precipitates	Anterior Chamber Inflammatory Cells	Iris Nodules	Angle Nodules	Clumped Vitreous Opacities	Diffuse Vitreous Opacities	Retinal Periphlebitis	Perivascular Nodules	Candle Wax Drippings (Retinochoroidal Exudates)	Optic Disc Granuloma	Choroidal Granuloma	Tent-Shaped or Trapezoidal Peripheral Anterior Synechiae	Photocoagulation-like Retinochoroidal Atrophic Lesions
3		Yes				Yes	Yes			Yes			
4					Yes	Yes			Yes				
5		Yes			Yes	Yes	Yes	Yes	Yes		Yes		Yes
6	Yes	Yes	Yes	Yes	Yes	Yes		Yes	Yes			Yes	Yes
9		Yes			Yes		Yes		Yes			Yes	
10	Yes	Yes		Yes	Yes	Yes	Yes	Yes	Yes			Yes	

**Table 4:** Treatments and outcomes.

Characteristic	Value
Topical corticosteroids	10 (100.0%)
Periocular corticosteroid injection (e.g., STTA)	7 (70.0%)
Systemic corticosteroids	5 (50.0%)
Cyclosporin	1 (10.0%)
Adalimumab	2 (20.0%)
Uveitis recurrence	7 (70.0%)
Uveitis-related glaucoma	8 (80.0%)
Glaucoma related to sarcoidosis or uveitis	7 (70.0%)
Steroid glaucoma	5 (50.0%)
Cataract surgery	4 eyes (20.0%)
Glaucoma surgery	1 eyes (5.0%)
Vitreous surgery	3 eyes (15.0%)
Extraocular inflammation: skin	7 (70.0%)
Extraocular inflammation: axillary lymph nodes	1 (10.0%)
Extraocular inflammation: unknown/none documented	2 (20.0%)

Note: Glaucoma was defined as the use of IOP-lowering medication and/or a history of glaucoma attack/surgery recorded in the registry. STTA: sub-Tenon’s triamcinolone acetonide injection.

**Table 5:** Patient-level treatments and outcomes.

Case	Periocular Steroid	Oral Steroid	Cyclosporin	Adalimumab	Recurrence	Glaucoma/ IOP Therapy	Chronic Inflammation	Steroid Glaucoma
1	Yes	Yes	No	Yes	Yes	Yes	Yes	Yes
2	No	No	No	No	No	Yes	Yes	Yes
3	Yes	No	No	No	No	No	-	-
4	Yes	No	No	No	Unknown	No	-	-
5	No	Yes	No	No	Yes	Yes	Yes	No
6	Yes	No	No	No	Yes	Yes	Yes	Yes
7	Yes	Yes	No	No	Yes	Yes	Yes	Yes
8	Yes	Yes	No	Yes	Yes	Yes	Yes	No
9	Yes	No	No	No	Yes	Yes	Yes	No
10	Yes	No	Yes	No	Yes	Yes	No	Yes

## Data Availability

The original contributions presented in this study are included in the article. Further inquiries can be directed to the corresponding author.
